# Response Surface Methodology and Artificial Neural Network Modelling of Membrane Rotating Biological Contactors for Wastewater Treatment

**DOI:** 10.3390/ma15051932

**Published:** 2022-03-04

**Authors:** Muhammad Irfan, Sharjeel Waqas, Ushtar Arshad, Javed Akbar Khan, Stanislaw Legutko, Izabela Kruszelnicka, Dobrochna Ginter-Kramarczyk, Saifur Rahman, Anna Skrzypczak

**Affiliations:** 1 Electrical Engineering Department, College of Engineering, Najran University Saudi Arabia, Najran 11001, Saudi Arabia; miditta@nu.edu.sa (M.I.); srrahman@nu.edu.sa (S.R.); 2 Chemical Engineering Department, University Teknologi PETRONAS, Seri Iskandar 32610, Malaysia; ushtar_18003307@utp.edu.my; 3 School of Chemical Engineering, The University of Faisalabad, Faisalabad 37610, Pakistan; 4 Mechanical Engineering Department, University Teknologi PETRONAS, Seri Iskandar 32610, Malaysia; 5 Faculty of Mechanical Engineering, Poznan University of Technology, 60-965 Poznan, Poland; stanislaw.legutko@put.poznan.pl; 6 Department of Water Supply and Bioeconomy, Faculty of Environmental Engineering and Energy, Poznan University of Technology, 60-965 Poznan, Poland; izabela.kruszelnicka@put.poznan.pl (I.K.); dobrochna.ginter-kramarczyk@put.poznan.pl (D.G.-K.); 7 Health-Fire-Environmental Specialist AIGO-TEC Sp. z o.o., Gnieźnieńska 6, 62-330 Nekla, Poland; a.skrzypczak@aigo-tec.com

**Keywords:** artificial neural networks (ANN), attached growth process, biofilm, response surface methodology (RSM), membrane fouling

## Abstract

Membrane fouling is a major hindrance to widespread wastewater treatment applications. This study optimizes operating parameters in membrane rotating biological contactors (MRBC) for maximized membrane fouling through Response Surface Methodology (RSM) and an Artificial Neural Network (ANN). MRBC is an integrated system, embracing membrane filtration and conventional rotating biological contactor in one individual bioreactor. The filtration performance was optimized by exploiting the three parameters of disk rotational speed, membrane-to-disk gap, and organic loading rate. The results showed that both the RSM and ANN models were in good agreement with the experimental data and the modelled equation. The overall R^2^ value was 0.9982 for the proposed network using ANN, higher than the RSM value (0.9762). The RSM model demonstrated the optimum operating parameter values of a 44 rpm disk rotational speed, a 1.07 membrane-to-disk gap, and a 10.2 g COD/m^2^ d organic loading rate. The optimization of process parameters can eliminate unnecessary steps and automate steps in the process to save time, reduce errors and avoid duplicate work. This work demonstrates the effective use of statistical modeling to enhance MRBC system performance to obtain a sustainable and energy-efficient treatment process to prevent human health and the environment.

## 1. Introduction

Membrane fouling that can result in the rapid decline of membrane flux is a major bottleneck for limiting the wide application of various membrane technologies [1]. Various methods to curtail membrane fouling are well developed, and many conventional and modern approaches to alleviate membrane fouling are in practice [2]. Conventional approaches focus on improving membrane properties, optimizing operational parameters, and tweaking the hydrodynamics near the membrane surface [3,4,5,6]. However, all these techniques result in high initial cost and high energy demand, thus limiting their widespread application [7,8]. The requirement for extensive coarse bubble aeration for fouling control in aerobic membrane bioreactors (MBR) imposes a high energy input of up to 50% of the total energy [9]. The high energy constraints to mitigate membrane fouling have been used as a basis for the development of low energy processes, such as vibrating and rotating disk MBR [10,11], suspended bio carriers in a moving bed biofilm reactor [12], hydrophilic modified MBR [13], and integrated fixed-film activated sludge MBR [14].

Response surface methodology (RSM) is an empirical statistical technique that can investigate mathematical modeling to comprehend the mutual relationship of various process parameters on the response variable. The quantitative data generated from the design of experiments and the analysis of regression models and operational conditions can result in high-end performance [15]. An artificial neural networks (ANN) is a statistical technique used as a predictive tool to develop a model that can forecast the outcome variable with a defined combination of input variables. The ANN is an emerging machine-learning tool due to its precise estimations of complex nonlinear systems [16]. Moreover, gravitational techniques have been also used to enhance separation performance [17,18,19].

Biological wastewater treatment incorporating membrane separation has been the focus of research worldwide [20,21,22]. The conventional activated sludge (CAS) process has been successfully incorporated with membrane separation technology to enhance overall performance in form of hybrid system of MBR. MBRs have gained popularity as advanced wastewater treatment technology that surpass the efficiency of the CAS process [23,24]. MBRs pose numerous advantages over the CAS process [25,26], which has led to their emerging full-scale implementation, particularly for water reuse purposes. However, the inherent drawbacks of membrane fouling in MBRs has not yet been completely addressed, which leads to high energy input [27]. The requirement for extensive coarse bubble aeration for fouling control in aerobic MBRs imposes high energy input cost of up to 50% of the total energy used [28].

Some studies have focused on the optimization of the operating conditions of the membrane process. Askari et al. [29] studied RSM to examine the effect of process conditions on NF membrane removal efficiency. The operational parameters of disk rotational speed, hydraulic retention time (HRT), and sludge retention time (SRT) significantly influence microbial community concentration, biological performance, and membrane fouling propensity [30,31]. Disk rotational speed, HRT, and SRT can alter the extracellular polymeric substance secretion, sludge settling characteristics, and mixed liquor properties [32,33]. When applied to wastewater treatment, RSM can describe the complex relationship between various operating parameters and optimize them by considering the response function linked to the overall performance evaluation of the wastewater treatment.

ANNs have been extensively used to predict effluent wastewater components in recent years. Aber et al. [34] utilized feed-forward backpropagation ANN modeling to predict the effluent Cr(VI) concentration in synthetic and real wastewater. The developed model gave a high correlation coefficient (R^2^ = 0.976), which indicates that the predicted model was successful. Pendashteh et al. [35] applied an ANN to optimize effluent chemical oxygen demand (COD), total organic carbon, and oil and grease concentration in the treatment of hypersaline oily wastewater in a membrane sequencing batch reactor. ANN modeling has also been used in a full-scale wastewater treatment plant to optimize the dynamics of the biological effluent characteristics (COD, biological oxygen demand, total nitrogen (TN), suspended solids (SS)) [36]. In previous research, ANNs have been employed to predict the membrane fouling potential and the evolution of hydraulic resistance and foulant thickness. Geissler et al. [37] established an ANN model to predict the membrane flux in a pilot-scale MBR by considering transmembrane pressure, SRT, filtration cycle, backwash cycle, total suspended solids, oxygen decay, and temperature.

The membrane rotating biological contactor (MRBC) developed in our previous study [38] diminishes membrane fouling through hydrodynamic adjustments. The membrane was placed inside the bioreactor between two rotating disks to scour the foulants from the membrane surface. The current research compares various machine learning and statistical approaches to maximize membrane permeability under the combined effect of disk rotational speed, membrane-to-disk gap, and organic loading rate. The optimization of operating parameters will help to ensure diminished membrane fouling. In addition, experimental measurements for the evaluation of membrane permeability could lead to the robustness of the decentralized wastewater treatment processes.

This study utilized RSM and ANN modeling and optimization techniques to investigate the relationship between variables by establishing the predicted models. This study investigates the relationship between operational parameters (disk rotational speed, membrane-to-disk gap, and organic loading rate) and the response parameter of permeability to find the optimal condition of the process. During experimentation, different operational parameters (disk rotational speed, membrane-to-disk gap, and organic loading rate) values were altered through a variable speed shaft motor and increases to the organic loading rate and sludge wastage rate, respectively, and the performance of the MRBC was analyzed. The optimization of the membrane-incorporated wastewater treatment process improved membrane permeability and reduced the operational cost of the process.

## 2. Materials and Methods

### 2.1. Wastewater Preparation

The synthetic wastewater was prepared by blending refined food leftovers (1 g/L) as suggested in previous work [39]. After mixing food leftovers with water, the mixture was left for 2 h to settle the suspended particles. The stock solution (supernatant) was then filtered through Whatman filter paper, 11 µm medium flow filter paper (Grade 1 Qualitative Filter Paper Standard Grade, GE Whatman, Kent, United Kingdom). The stock solution was then diluted to obtain the influent wastewater concentration as summarized in Table 1. The prepared wastewater was analyzed in terms of COD, TN, ammonium, and nitrate.

### 2.2. Bioreactor Set-Up and Operation

**Figure 1:**
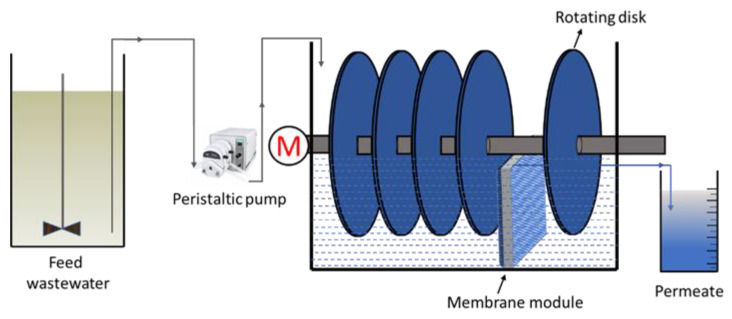
Schematic diagram of the membrane rotating biological contactor configuration.

The MRBC bioreactor was fabricated in-house using acrylic based material and operated in accordance with the layout depicted in Figure 1. The bioreactor consisted of a 45 L storage tank and a 6.5 L bioreactor. A total of 5 disks fabricated from methyl methacrylate sheets with an 18 cm diameter were attached to a stainless-steel shaft in the bioreactor. To colonize the microbial population, the disks were attached to polyurethane sheets (1.22–1.27 g/cm^3^ density). The polyurethane-coated disks, 3 cm apart from each other (corresponding to a net surface area of 2034 cm^2^), were placed inside the MRBC bioreactor at 40% submergence. The feed wastewater from the storage tank was constantly pumped, with a peristaltic pump, to the bioreactor. The bioreactor with 25 × 25 × 30 cm^3^ dimensions was fabricated in-house with methyl methacrylate sheets. The flat-sheet membrane module was placed inside the bioreactor between two rotating disks. The MRBC system does not include the settling tank part of a conventional RBC unit. The detailed experimental procedure, equipment specifications, and membrane fabrication procedure for the MRBC bioreactor can be seen in the previously published article [38].

The membrane sheet was cut and fixed onto both sides of the panel, forming a plate and frame filtration panel. The membrane sheet that was attached to a panel had a semi-circular shape and resulted in an active membrane surface area of 226 cm^2^. The membrane sheets were glued to the panel with A-B epoxy glue (A-B quick epoxy, HYRO, Kuala Lumpur, Malaysia). The filtration panel was confirmed to be free from leakage. A spacer fabric that was placed between the two membrane sheets acted as a permeate channel. The membrane permeate was evacuated through a permeate pipe that connected the permeate channel to the permeate pump.

The bioreactor was run for 42 days, divided into two periods. During the first 15-day period, the bioreactor was operated under constant loading conditions of 10 g COD/m^2^ d to grow and acclimatize the biofilm atop the polyurethane foam surface. During this period, the biofilm was observed carefully, and biological performance was monitored regularly. After the acclimatization phase, the biofilm was completely developed and was effective at degrading organics and nutrients. Any detached flocs from the rotating disks were regularly discharged. At this stage, the membrane panel was installed inside the RBC bioreactor and the effects of the operational parameters on membrane fouling was observed.

### 2.3. Analytical Methods

COD, TN, ammonium, and nitrate were measured using the specific Hach digestion solution (HACH, Loveland, CO, USA) for each compound. The solution was diluted to fall into the range of the digestion vials being used for the study. The values were determined through a Hach DR3900 Spectrophotometer (HACH, Loveland, CO, USA). A Hach 2100Q portable turbidimeter (HACH, Loveland, CO, USA) and a Hach HQ411D benchtop PH/MV meter (HACH, Loveland, CO, USA) were used to determine the turbidity and the pH, respectively [40].

### 2.4. Experimental Design Using the Response Surface Methodology Method

RSM is an aggregation of mathematical and statistical approaches to examine the effectiveness of various operational parameters. Design-Expert software, Suite, MN, USA (DES) version 8.0.6 was applied to evaluate the responses of multiple parameters [41,42]. The application of RSM to design optimization reduces the cost of otherwise-expensive analysis methods and their associated numerical noise. The response variable can be represented graphically (contour plots or three-dimensional space) to help visualize the response surface shape. The RSM principle is based on two fundamental concepts: selecting the approximate model and evaluating the response. The selection of an approximate model is helpful to obtain the optimized solution at the expense of minimum experimentation. The objective of DOE is the selection of the points where the response should be evaluated.

To scrutinize the impact of various operating parameters on the membrane fouling propensity in the MRBC configuration, three numerical factors with a 5-level CCD model were employed, holding 13 central points per block. In a 5-level CCD, each numeric factor is varied over five levels: the center point, plus and minus alpha (axial points), and plus and minus 1 (factorial points). The three independent operational parameters selected were (i) disk rotational speed, (ii) membrane-to-disk gap, and (iii) organic loading rate. The CCD consisted of 5 levels: high level or maximum (referred to as +1), medium level or central (referred as 0), low level or minimum (referred as −1), and plus and minus alpha, for all operating parameters. The disk rotational speed varied from 30–50 rpm. The membrane-to-disk gap was 1–3 cm, while the organic loading rate was changed from 10–30 g COD/m^2^ d, as shown in Table 2.

### 2.5. Artificial Intelligence and Machine Learning Approach

**Figure 2:**
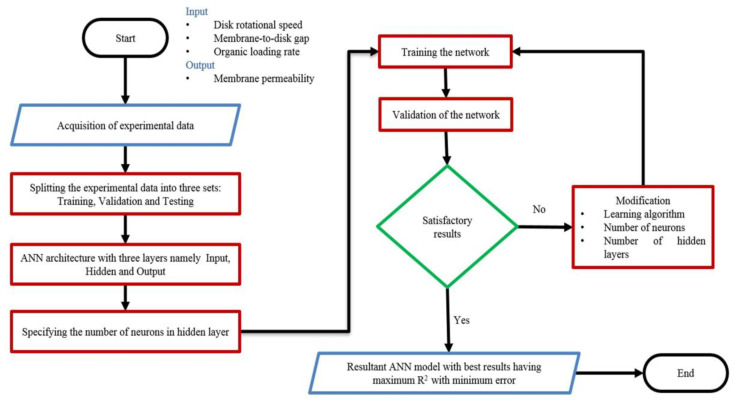
Process flow of artificial neural networks modelling.

Artificial intelligence and machine learning approaches for optimization problems have gained popularity among researchers due to their sufficiently accurate results. A process flow for the development of the ANN model is given in Figure 2. The proposed ANN model took three input factors, i.e., disk rotational speed, membrane-to-disk gap, and organic loading rate, with membrane permeability being the output variable. The experimental data was divided into training (70%; 39 samples), validation (15%; 8 samples), and testing (15%; 8 samples) sets. The number of hidden layers and hidden neurons of the trained ANN model substantially impacts its predictive performance. Therefore, carefully choosing the number of hidden layers and hidden neurons is critical [43]. MATLAB^®^ 2020b was utilized in this research to develop the FFBPN-based ANN model to predict the response. The developed model was trained until satisfactory results were obtained. The best-optimized model was selected based on the highest R-squared value, and lowest mean squared error (MSE).

## 3. Results and Discussion

### 3.1. Statistical Analysis and Model Development

Design of experiment (DOE) was used to design 55 experimental runs. The results of experiments in terms of permeability are shown in Table 3. A central composite design (CCD) matrix model was applied to predict the permeability in each experimental run.

### 3.2. RSM Model Optimization

A full-fractional, three factorial CCD was applied to examine the effects of three independent parameters to model the steady-state membrane permeability. The model analysis results show that the quadratic model was significant, as the R^2^ was high (0.9762) and the probability values were low (*p* ≤ 0.0001). According to the results of the quadratic model, 8 out of 9 model terms were significant (*p* ≤ 0.05). The coded and actual factors for membrane permeability are shown in Equations (1) and (2).

Steady-state membrane permeability (L/m^2^ h bar) = 297.10 + 6.65 A − 10.00 B − 2.40 C − 3.06 AB + 1.19 AC + 1.98 BC − 12.72 A^2^ − 3.61 B^2^ + 0.3632 C^2^ (1)

Steady-state membrane permeability (L/m^2^ h bar) = 71.72939 + 11.21426 × Disk rotational speed + 12.74987 × Membrane-to-disk gap − 1.25576 × Organic loading rate − 0.306250 × Disk rotational speed × Membrane-to-disk gap + 0.011875 × Disk rotational speed × Membrane-to-disk gap + 0.197917 × Membrane-to-disk gap × Organic loading rate − 0.127182 × Disk rotational speed^2^ − 3.61425 × Membrane-to-disk gap^2^ + 0.003632 × Organic loading rate^2^ (2)

The significant model terms were disk rotational speed (A), membrane-to-disk gap (B), and organic loading rate (C), the square terms of disk rotational speed (A^2^) and membrane-to-disk gap (B^2^), and the interaction terms of AB, AC, and BC (Table 4). The insignificant term square of organic loading rate (C^2^) was removed from the final equations. The authenticity and significance of the model were calculated based on different constraints. The R^2^ value determines the quality of fitness of the model. An R^2^ close to 1 signifies the good quality of the model, while *p* ≤ 0.05 defines the significance of the proposed model. In the current study, the R^2^ for permeability was 0.9762 while Adj.R^2^ was 0.9714, respectively. For a good fitness of model, R^2^ should be higher than 0.8, and an R^2^ close to 1 suggests great accordance between the experimental data and the proposed model data.

**Figure 3:**
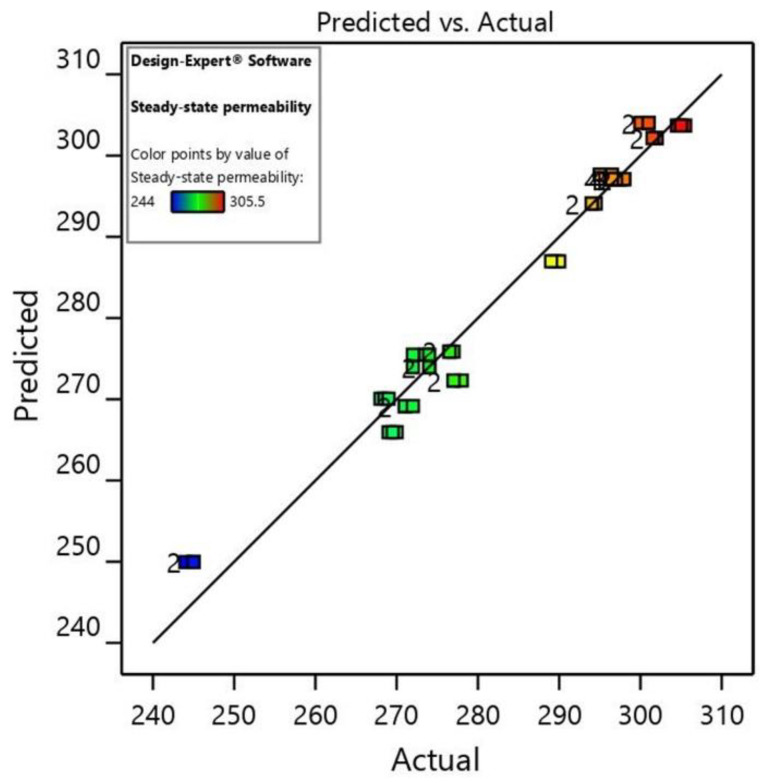
Design-Expert plot of predicted vs. actual values plot for permeability.

The model precision can be assessed using a diagnostic diagram of predicted vs. actual values. Figure 3 shows the plot of predicted vs. actual permeability values. All the experimental points lie near the straight line, indicating the consistency of a normal distribution. A good correlation between the actual and predicted values for the response function confirms the adequacy of the proposed model.

### 3.3. Process Analysis

**Figure 4:**
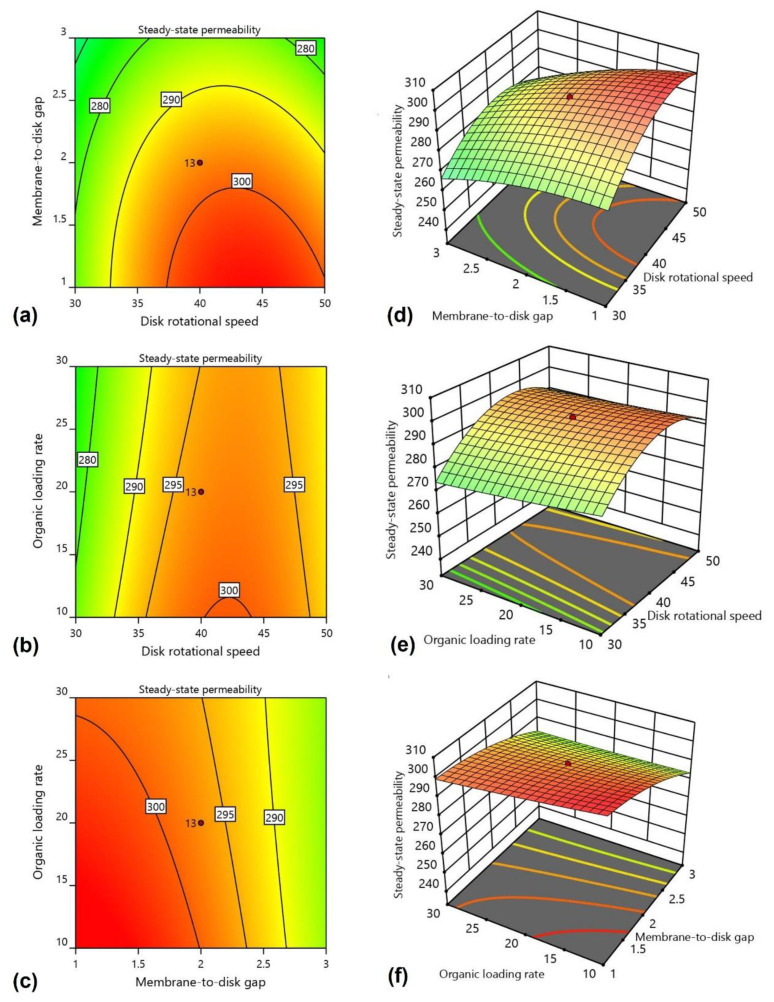
Effect on 2-D contours of (**a**) disk rotational speed and membrane-to-disk gap, (**b**) disk rotational speed and organic loading rate, and (**c**) membrane-to-disk gap and organic loading rate; and 3-D response surface plots of (**d**) disk rotational speed and membrane-to-disk gap, (**e**) disk rotational speed and organic loading rate, and (**f**) membrane-to-disk gap and organic loading rate.

Figure 4 shows the effects of the operating parameters (disk rotational speed, membrane-to-disk gap, and organic loading rate) on the permeability in 2-D contour and 3-D response surface plots.

Figure 4a,d demonstrate the effects of disk rotational speed and membrane-to-disk gap on the permeability. In these conditions, the organic loading rate was kept constant at 20 g COD/m^2^ d. The results demonstrate that with the increase to disk rotational speed, permeability increases because higher shear is generated with increased speed, which removes the deposited foulants from the membrane surface. However, it can be seen from the figures that, after 45 rpm, the value of permeability decreases. The decline in permeability was due to a higher shear rate, which shreds the biofilm layer from the rotating disks. The shredded layer remains suspended as biofilm floc and does not settle easily. The shredded biofilm layer is deposited at the membrane surface, blocking the membrane pores and decreasing its permeability [44,45,46]. The optimum disk rotational speed for the maximum permeability was around 45 rpm. A less critical effect was observed for membrane-to-disk gap than disk rotational speed on the permeability. As the membrane-to-disk gap decreases from 3 cm to 1 cm, the permeability increases because the membrane’s placement near the disks results in a higher shear being induced at the membrane surface. This result indicates that a small membrane-to-disk gap results in higher membrane permeability.

Figure 4b,e show the effect of disk rotational speed and organic loading rate on the permeability when the membrane-to-disk gap was kept constant at 2 cm. The organic loading rate, which varied from 10 to 30 g COD/m^2^ d, did not significantly impact the system’s overall performance. The influent wastewater was readily degraded by the microorganisms present in the bioreactor. The previous study showed results indicating a higher biological performance efficiency at a lower hydraulic retention time. The lower the organic loading rate, the higher the permeability due to efficient microbial activity.

The interaction between the membrane-to-disk gap and organic loading rate is shown in Figure 4c,f. Both the membrane-to-disk gap and the organic loading rate increases were directly proportional to permeability. A rise in permeability was observed at lower membrane-to-disk gaps and higher organic loading rates. Indeed, the membrane permeability increases further at higher organic loading rates. Overall, high values of organic loading rate and minimum membrane-to-disk gaps favor higher permeability. Our results showed higher permeability at around the 10 g COD/m^2^ d organic loading rate and with a 1 cm membrane-to-disk gap, respectively.

The results indicate that membrane fouling was significantly dependent on the operating parameters. Optimal membrane permeability was found at higher disk rotational speeds, lower membrane-to-disk gaps, and higher organic loading rates. Higher microbial community growth facilitated the decomposition of the substrate at higher loading rates. The selection of optimized operating conditions can help obtain higher permeability with minimum energy demand.

### 3.4. Process Optimization

The optimization of membrane permeability under the operating conditions was performed through process optimization. The highest permeability value was selected under the optimum operational parameters to optimize the response function. Table 5 shows that the maximum permeability value of 308.4 L/m^2^ h bar was obtained at the 44 rpm disk rotational speed, the 1.07 cm membrane-to-disk gap, and the 10.2 g COD/m^2^ d organic loading rate, respectively. Under these conditions, the degree of desirability of the model was equal to 1.

Two more experiments were performed using the optimum operating conditions to confirm the achieved results from the experiments and model (Table 6). The experimental and model membrane permeability obtained from these optimum values were in close agreement, verifying the precision of the developed model.

### 3.5. Artificial Neural Networks (ANN)

The statistical modeling approach, ANN, was used to improve the accuracy and reliability of the predicting process. An ANN establishes a complex correlation among independent and dependent variables and may replace traditional multiple regression modeling techniques [47]. During the network’s training phase, the Levenberg–Marquardt algorithm was suitable for the trained network. The ANN model was trained based on the feed-forward backpropagation iterative method in MATLAB^®^ 2020b. The sigmoid function ”tansig” and the linear activation function ”purlin” were used in the hidden and output layers. The ”tansig” activation function is given in Equations (3) and (4) [47].

(3) $$f\left(x\right)=\frac{e^{x}-e^{-x}}{e^{x}+e^{-x}}$$

(4) $$x_{j}=\overset{N}{\underset{i=1}{\sum }}w_{ij}y_{i}+b_{j}$$

where *x* is the weighted sum of the inputs calculated using weights (*w*), biases (*b*), and outputs (*y_i_*).

**Figure 5:**
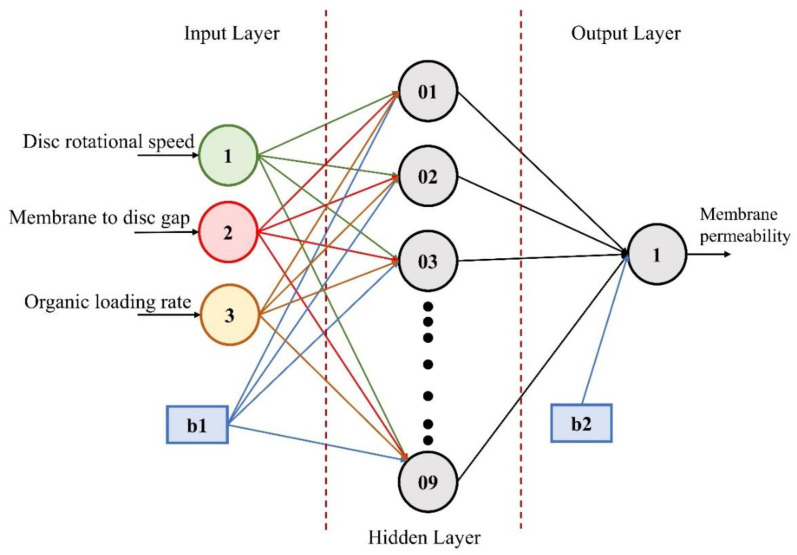
The architecture of the trained artificial neural networks model.

Various topological networks have been tried by changing the hidden layer neurons to optimize the network. However, for this study, the trained network with the highest accuracy and robust predictive ability was found with the architecture of 3-9-1, as given in Figure 5.

**Figure 6:**
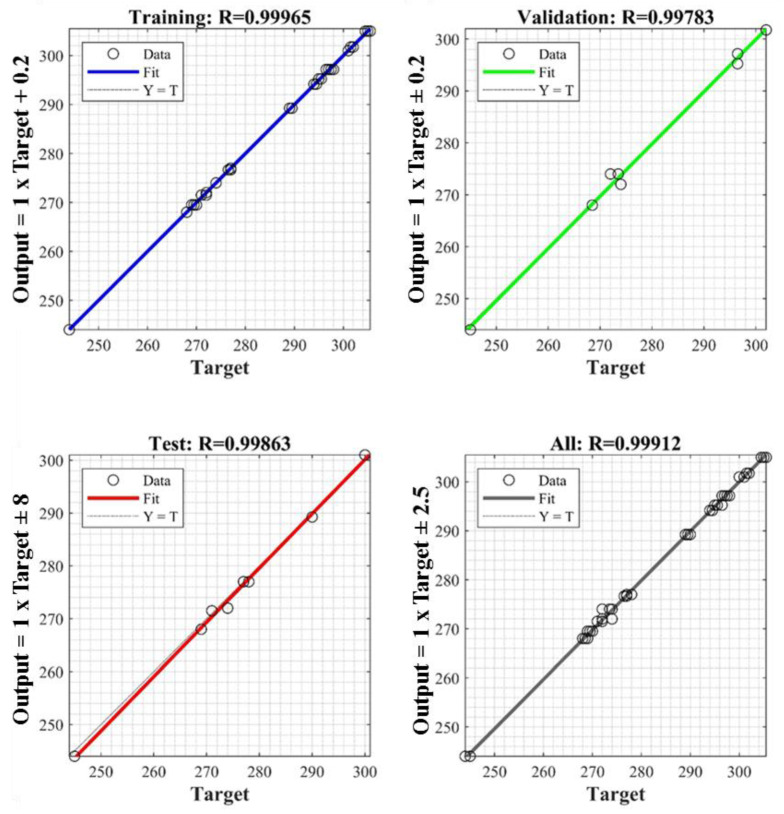
Regression plots for R values with overall R-squared = 0.99824.

The regression plots for the developed model for the training, testing, and validating models are provided in Figure 6. A good correlation can be observed between the actual data and the values predicted by the model. The model’s performance was also investigated for the validating and testing datasets of experimental data. Figure 6 shows the actual vs. predicted values for the training, validation, testing, and comprehensive data sets with R^2^ values of 0.99965, 0.99783, 0.99863, and 0.99912, respectively. Furthermore, the obtained mean squared error (MSE) values were 0.137, 1.44, 1.10, and 0.468 for training, validation, testing, and overall model development, respectively. These results show that the anticipated and test values were highly correlated. The higher R^2^ values and lower MSE values ensured the robustness of the trained model [48]. The results show that the developed network was adequately capable of learning the relationship between the input and output parameters and, therefore, could be applied to predict the optimal operating conditions for the process.

The RSM and ANN models were compared based on the statistical performance indices given in Equations (5)–(7) to highlight the predictive ability of the developed models. The coefficient of determination R^2^ measures how much of the overall statistical variance in the observed dataset can be explained by the model, as given in Equation (5). The MSE given in Equation (6) is the average squared difference between the estimated values and the actual values. A measure of the overall credibility of the entire spectrum of the dataset is calculated using the Root Mean Square Error (RMSE). A squared scale given in Equation (7) makes it responsive to small changes in model outcomes while also exhibiting strong sensitivities to larger errors at higher magnitudes [2].

(5) $$R^{2}={\left(\frac{\sum_{i=1}^{N}(L_{i}^{Pred}-L_{i}^{Exp})(L_{i}^{Pred}-L_{i}^{Exp})}{\sqrt{\sum_{i=1}^{N}{\left(L_{i}^{Exp}-L_{i}^{Exp\;Mean}\right)}^{2}\sqrt{\sum_{i=1}^{N}{\left(L_{i}^{Pred}-L_{i}^{Pred\;Mean}\right)}^{2}}}}\right)}^{2}$$

(6) $$MSE=\frac{1}{N}\overset{N}{\underset{i=1}{\sum }}{\left(L_{i}^{Exp}-L_{i}^{Pred}\right)}^{2}$$

(7) $$RMSE=\sqrt{\frac{\sum_{i=1}^{N}{\left(L_{i}^{Exp}-L_{i}^{Pred}\right)}^{2}}{N}}$$

where *L^Exp^* is experimental permeability, *L^Pred^* is predicted permeability *L^Exp Mean^* is the mean of the experimental values, and *L^Pred Mean^* is the mean of the predicted permeabilities.

The values for these performance indices are given in Table 7. When comparing the two models, it was evident, based on the statistical indices, that the ANN model outperformed the RSM model in prediction capabilities. A higher R^2^ value was obtained for the ANN (0.9982) than the RSM (0.9762). An R^2^ value closer to 1 indicates a good correlation between the actual and predicted values. The MSE and RMSE values are also lower for the ANN compared to RSM. This parameter value suggests that the ANN model was more efficient than RSM in prediction, and therefore that it could predict more accurately the input and output parameters of the model.

The motions of the disks in the MRBC system induce the secondary flow of liquid dragged by the disk rotation that scours off foulant effectively from the membrane surface. As a result, the MRBC had a higher permeability, a very promising result confirming the effectiveness of the hybridization of RBC and membrane filtration. The distinct performances of the MRBC indicate the positive impact of hydrodynamics in membrane fouling control. The membrane panel in the MRBC was positioned much closer to the rotating disk and it thus experienced a higher shear rate. In another study, 34.6% higher steady-state permeability was obtained by exploiting the hydrodynamics near the membrane surface by the simple projection of air bubbles using a finned spacer [49]. Vibrating the membrane also demonstrates the effectiveness of the shear rates for the removal of macromolecules, colloids, and other foulants from the membrane surface [10]. These studies utilized energy-intensive coarse bubble aeration and membrane vibration to generate the shear rates and to impose membrane fouling control.

The rate of rotational speed was, however, limited, to avoid the “seeding effect”, a phenomenon of biofilm detachment due to centrifugal forces [50]. The highest value of steady-state permeability observed was at a rotational speed of 44 rpm. Unlike other rotary disk systems that use a separate membrane fouling control (which is another energy consuming factor, as noted by [4]), the MRBC configuration utilizes the existing disk rotation in the conventional RBC for membrane fouling control and thus does not consume extra energy. However, the optimal adjustment of the disk rotational speed is required because the application of higher rotational speed may result in higher permeability, but enhanced shear rates may cause media slaughtering (also known as the seedling effect) which promotes the development of suspended biofilm, and therefore membrane fouling.

The positive effect of hydrodynamics on membrane fouling control, as demonstrated in this study, were extensively been reported for MBRs [51] either via liquid secondary flow or through the addition of cleaning media, which confirmed our results. The application of a rotary disk in combination with sponge media enhanced filtration performance in an anaerobic membrane bioreactor [52]. A sponge-like carrier media in MBR improved permeability in comparison to a conventional submerged MBR due to its media scouring impact and carrier circulation [53]. Kim at al. [54] reported that membrane fouling in an anaerobic fluidized bed bioreactor could be effectively controlled by the addition of granular activated carbon.

## 4. Conclusions

Adjusting the hydrodynamics near the membrane surface can significantly dampen membrane fouling propensity. This study demonstrated an MRBC system to control membrane fouling through the generation of a certain shear rate near the membrane surface. Membrane optimization is important role because it helps to reduce costs and can lead to higher profitability. An RSM and an ANN were applied for optimizing membrane permeability through its operating parameters. The RSM suggested a quadratic model for the prediction of permeability. ANOVA analysis indicated that all three operating parameters (disk rotational speed, membrane-to-disk gap, and organic loading rate) significantly impact the permeability. The results indicated optimum values of a 44 rpm disk rotational speed, a 1.07 cm membrane-to-disk gap, and a 10.2 g COD/m^2^ d organic loading rate would produce the highest membrane permeability of 308.4 L/m^2^ h bar. An ANN was applied to investigate the potential of a feed-forward backpropagation network to predict membrane permeability. An overall R^2^ of 0.9982 was obtained for the optimized network, while the RSM resulted in an R^2^ value of 0.9762. This work demonstrated the effective use of RSM and ANN modelling techniques to optimize operational parameters. The findings showed good agreement between the experimental data and the predicted equation. Therefore, both RSM and ANN can be proposed as valuable tools for optimizing wastewater treatment processes.

## Figures and Tables

**Table 1:** Characteristics of the influent wastewater.

Sr #	Contaminant	Unit	Concentration
1	COD	mg/L	298 ± 45.6
2	TN	mg/L	2.4 ± 0.2
3	Ammonium	mg/L	0.92 ± 0.07
4	Nitrate	mg/L	0.52 ± 0.08
5	Turbidity	NTU	15.2 ± 0.6
6	pH	--	6.35 ± 0.18

COD: chemical oxygen demand, TN: total nitrogen.

**Table 2:** Independent variables and levels used in central composite design.

Levels	Independent Variable	Low Level (−1)	Medium Level (0)	High Level (+1)
1	Disk rotational speed	30	40	50
2	Membrane-to-disk gap	1	2	3
3	Organic loading rate	10	20	30

**Table 3:** Design of experimental runs for the independent variables and response functions.

Run	Independent Variables			Permeability (L/m^2^ h bar)	
	Disk Rotational Speed (rpm)	Membrane-to-Disk Gap (cm)	Organic Loading Rate (g COD/m^2^ d)	Actual Value	Predicted Value
1	50	3	10	274	273.95
2	40	2	20	296.5	297.10
3	50	1	10	300	304.03
4	40	2	20	298	297.10
5	40	2	20	297	297.10
6	30	1	30	276.5	275.86
7	30	3	30	269	265.95
8	50	3	10	274	273.95
9	40	2	37	294	294.10
10	57	2	20	277	272.31
11	30	1	10	289.5	286.99
12	23	2	20	245	249.95
13	50	3	30	273.5	275.49
14	40	2	20	297.5	297.10
15	40	0.3	20	305	303.70
16	40	2	20	296.5	297.10
17	50	3	30	274	275.49
18	30	1	10	289	286.99
19	40	2	20	298	297.10
20	40	3.7	20	268	270.07
21	40	2	3.2	302	302.16
22	40	2	37	294	294.10
23	40	2	20	297	297.10
24	30	3	10	271	269.16
25	57	2	20	277	272.31
26	50	1	30	295	297.66
27	40	2	20	297.5	297.10
28	40	2	37	294.5	294.10
29	30	1	10	290	286.99
30	30	3	10	271	269.16
31	40	0.3	20	304.5	303.70
32	50	3	30	272	275.49
33	50	3	10	272	273.95
34	30	3	30	269.5	265.95
35	30	3	30	270	265.95
36	40	2	3.2	301.5	302.16
37	30	1	30	277	275.86
38	40	3.7	20	269	270.07
39	57	2	20	278	272.31
40	23	2	20	244	249.95
41	50	1	30	295.5	297.66
42	50	1	10	301	304.03
43	30	3	10	272	269.16
44	40	2	20	296.5	297.10
45	30	1	30	276.5	275.86
46	40	0.3	20	305.5	303.70
47	40	2	20	297	297.10
48	40	3.7	20	268.5	270.07
49	40	2	3.2	302	302.16
50	23	2	20	245	249.95
51	40	2	20	297.5	297.10
52	40	2	20	297	297.10
53	50	1	10	301	304.03
54	50	1	30	296.5	297.66
55	40	2	20	296.5	297.10

**Table 4:** ANOVA results of the coefficient of quadratic model for permeability.

Source	Sum of Squares	Df	Mean Square	*F*-Value	*p*-Value	Parameter Significance
Model	13,229.15	9	1469.91	204.66	<0.0001	Significant
A-Disk rotational speed	1809.98	1	1809.98	252.01	<0.0001	-
B-Membrane-to-disk gap	4096.07	1	4096.07	570.32	<0.0001	-
C-Organic loading rate	235.28	1	235.28	32.76	<0.0001	-
AB	225.09	1	225.09	31.34	<0.0001	-
AC	33.84	1	33.84	4.71	0.0353	-
BC	94.01	1	94.01	13.09	0.0007	-
A²	6316.25	1	6316.25	879.45	<0.0001	-
B²	510.08	1	510.08	71.02	<0.0001	-
C²	5.15	1	5.15	0.7173	0.4015	-
Residual	323.19	45	7.18	-	-	-
Lack of Fit	308.45	5	61.69	167.37	<0.0001	significant
Pure Error	14.74	40	0.3686	-	-	-
Cor Total	13,552.35	54				-
Other statistical parameters						
R^2^	Adjusted R^2^	S.D.	A.P.	C.V. (%)	-	-
0.9762	0.9714	2.68	47.3233	0.9396	-	-

**Table 5:** Optimized operational parameter values at maximum permeability.

Variables	Optimum Values	Steady-State Permeability (L/m^2^ h Bar)		Error (%)	Standard Deviation
		Predictive	Experimental		
Disk rotational speed	44 rpm	309	309.5	0.16	2.68
Membrane-to-disk gap	1.07 cm				
Organic loading rate	10.2 g COD/m^2^ d				

**Table 6:** Permeability response function for the experimental and model values.

	Steady-State Permeability (L/m^2^ h Bar)			
Run	Predictive	Experimental	Error (%)	Standard Deviation
1	143.5	143.00	0.35	0.26
2	137.3	137	0.18	0.13

**Table 7:** Comparison of response surface methodology and artificial neural networks models based on statistical performance indices.

Statistical Performance Index	RSM	ANN
R^2^	0.9762	0.9982
MSE	5.8709	0.4680
RMSE	2.4230	0.6840

## Data Availability

All the data is available within the manuscript.
